# Obscure Small Bowel Bleeding Related to a Ventral Hernioplasty Mesh Perforation Visualized With Video Capsule Endoscopy

**DOI:** 10.7759/cureus.60908

**Published:** 2024-05-23

**Authors:** Mei Yang, Biruk Beyene, Elie Chahla

**Affiliations:** 1 Internal Medicine, St. Luke's Hospital, Chesterfield, USA; 2 Gastroenterology, St. Luke's Hospital, Chesterfield, USA

**Keywords:** small bowel bleeding, video capsule endoscopy, abdominal hernia repair, mesh perforation, ventral hernioplasty

## Abstract

We report a case of a 76-year-old female presenting with intermittent obscure gastrointestinal (GI) bleeding originating from the small intestine secondary to a delayed complication related to mesh hernioplasty. The mesh was eroding into the small bowel causing intermittent transfusion-dependent GI bleeding. Multiple upper and lower endoscopic investigations were sought over the last two years, but they were noncontributory. Finally, video capsule endoscopy (VCE) revealed mesh invasion into the small bowel wall associated with bleeding. This case emphasizes the significance of an early sufficient differential diagnosis in patients with obscure GI bleeding. Meanwhile, being cognizant of rare causes of GI bleeding in patients who have had hernioplasty is very important.

## Introduction

Surgical hernioplasty is a very common procedure that treats different types of hernias like ventral hernias [1,2]. A mesh device is a surgical technique used to repair ventral hernias, which may cause possible serious complications including mesh migration, or improper placement of the mesh [3]. Small bowel obstruction, perforations, erosions, fistulas, and volvulus are complications after this type of hernia repair and have been attributed to mesh migration [2-4]. Imaging procedures, including endoscopy, colonoscopy, or abdominal computed tomography, are useful to locate or diagnose this disease [1,4]. Literature research revealed only one published case of obscure gastrointestinal (GI) bleeding due to mesh migration from ventral hernioplasty in the United States [1].

## Case presentation

**Figure 1:**
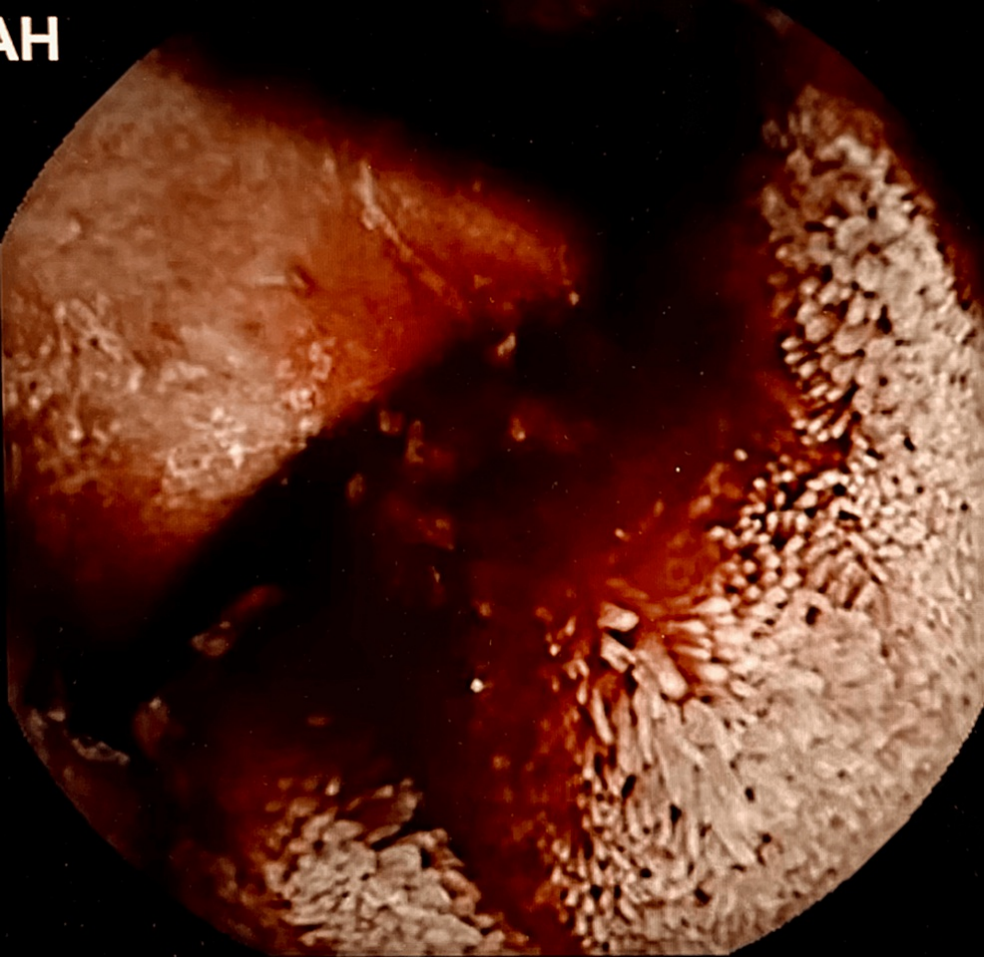
Video capsule endoscopy showing small bowel intraluminal bleeding.

**Figure 2:**
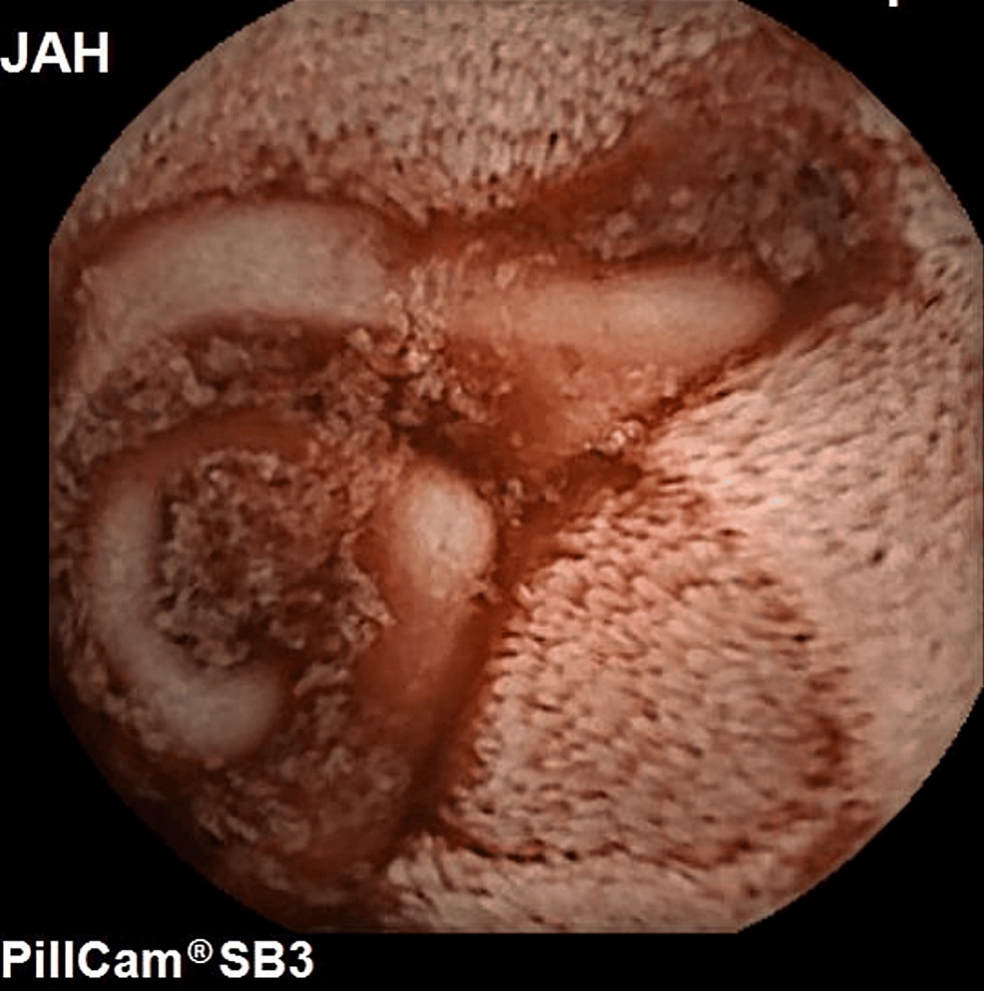
Video capsule endoscopy showing intraluminal coiled mesh eroding through wall of the jejunum into lumen.

A 76-year-old Caucasian female with a significant past medical history of obesity, Insulin-dependent diabetes mellitus, hypertension, dyslipidemia, chronic obstructive pulmonary disease (COPD), early-stage esophageal adenocarcinoma status post concurrent chemotherapy and radiation therapy with good response, complicated diverticulitis status post left hemicolectomy, pancreatitis, chronic blood loss anemia, open cholecystectomy with incisional hernia repair and persistent large ventral hernia, recurrent hospitalizations for anemia and hematochezia. Two times esophagogastroduodenoscopies (EGDs) and a one-time colonoscopy as part of her anemia workup were done recently. EGD showed the esophageal cancer was in remission, without any active bleeding. A colonoscopy revealed a benign-appearing nonobstructing inflammatory proximal rectal anastomotic stricture which was less likely as an etiology of her anemia. She was hemodynamically stable, and her hemoglobin was noted at 7.6 g/dL down from a baseline of 9 g/dL in the last year. Recent upper and lower endoscopies were just done several weeks ago, which showed no clear source of bleeding. A small bowel bleeding source needed to be excluded this time and a video capsule endoscopy (VCE) followed. This revealed evidence of a foreign body compatible with mesh perforating the lumen of the mid jejunum with associated small bowel bleeding (Figures 1, 2). Abdominal CT with intravenous contrast was done and failed to reveal the presence of perforation. Since it was not possible to extract the mesh or correct the bleeding endoscopically, she was transfused a total of three units of blood with stabilization of her hemoglobin. We consulted the surgical services. When presented with the potential complications linked to the surgery given her significant comorbidities, the patient refused additional interventions. She was eventually discharged home with a close hematology follow-up. She is still doing well with scheduled iron infusions and blood transfusions on an as-needed basis.

## Discussion

Recent literature reported that ventral hernia repair has various complications with the mesh technique, especially bowel perforation, peritonitis, and bowel obstruction from mesh migration [4,5]. The case we described here represents the second-ever published case of a ventral hernia mesh migration into the mid jejunum visualized with VCE in a patient presenting with melena [1].

VCE is a well-established diagnostic method for further small bowel investigation in obscure GI bleeds, according to endoscopic recommendations released by the American Society for Gastrointestinal Endoscopic. A VCE is a procedure in which a patient needs to swallow a capsule with a small wireless camera inside to take thousands of pictures as it passes through the small intestine. Compared to Push enteroscopy and CT angiography, VCE offers a higher yield for identifying bleeding sources and clinically relevant lesions in the small intestine. An uncommon complication of VCE is capsule retention (1.4%), which can cause bleeding, infection, or obstruction [6,7]. If capsule retention happens, close monitoring was suggested because capsule retention generally can resolve spontaneously by itself. However, if the conservative method fails, then a surgical approach would be the next step.

Our team determined that VCE was the best course of action because the patient had recurrent episodes of obscure GI bleeding after unrevealing upper endoscopy and colonoscopy. Thus, another essential diagnosis method VCE is very significant to find the bleeding source and etiology [7]. Meanwhile, the mesh is a large prosthetic hernia repair device that cannot be removed endoscopically without major complications, and surgery would be the next step consideration if the patient is unstable after transfusion. But, in our case, the elderly patient has many complicated medical histories and is not a good candidate for surgery.

## Conclusions

Mesh migration, a well-known side effect of surgical hernioplasty, frequently results in perforation or small bowel obstruction. GI bleeding from mesh migration is an uncommon and rare complication. This case highlights the significance of a thorough diagnostic examination in patients with obscure GI bleeding as well as the need to be aware of uncommon causes of GI bleeding in patients who have had hernioplasty.

## References

[1] Mendez-Ishizaki Y, Parra JL (2016). Obscure overt gastrointestinal bleeding secondary to ventral hernioplasty mesh small bowel perforation visualized with video capsule endoscopy. ACG Case Rep J.

[2] Agrawal A, Avill R (2006). Mesh migration following repair of inguinal hernia: a case report and review of literature. Hernia.

[3] LeBlanc KA (2001). Complications associated with the plug-and-patch method of inguinal herniorrhaphy. Hernia.

[4] Dieter RA Jr (1999). Mesh plug migration into scrotum: a new complication of hernia repair. Int Surg.

[5] Lo DJ, Bilimoria KY, Pugh CM (2008). Bowel complications after prolene hernia system (PHS) repair: a case report and review of the literature. Hernia.

[6] Wang A, Banerjee S, Barth BA (2013). Wireless capsule endoscopy. Gastrointest Endosc.

[7] Fisher L, Lee Krinsky M, Anderson MA (2010). The role of endoscopy in the management of obscure GI bleeding. Gastrointest Endosc.

